# Anti-malarial activity of geldanamycin derivatives in mice infected with *Plasmodium yoelii*

**DOI:** 10.1186/1475-2875-11-54

**Published:** 2012-02-23

**Authors:** Rubul Mout, Zhi-Dong Xu, Angela K H Wolf, Vincent Jo Davisson, Gotam K Jarori

**Affiliations:** 1 Department of Biological Sciences, Tata Institute of Fundamental Research, Homi Bhabha Road, Colaba, Mumbai 400005, India; 2 Department of Medicinal Chemistry & Molecular Pharmacology, Purdue University, West Lafayette, IN 47906, USA; 3 Bindley Bioscience Center, Purdue University, West Lafayette, IN 47906, USA

**Keywords:** *Plasmodium yoelii*, Geldanamycin, Immunity, Normocyte, Reticulocyte

## Abstract

**Background:**

Geldanamycin (GA), a benzoquinone ansamycin antibiotic has been shown in vitro to possess anti-plasmodial activity. Pharmacological activity of this drug is attributed to its ability to inhibit PfHSP90. The parasite growth arrest has been shown to be due to drug-induced blockage of the transition from ring to trophozoite stage. To further evaluate the consequences of this pharmacodyamic feature, the anti-malarial activity of GA analogs with enhanced drug properties in a *Plasmodium*-infected animal model have been evaluated for their capacity to induce clearance of the parasite. In the process, a hypothesis was subsequently tested regarding the susceptibility of the cured animals to malaria reflected in an attenuated parasite load that may be evoked by a protective immune response in the host.

**Methods:**

Six weeks old Swiss mice were infected with a lethal *Plasmodium yoelii *(17XL) strain. On appearance of clinical symptoms of malaria, these animals were treated with two different GA derivatives and the parasite load was monitored over 15-16 days. Drug-treated animals cured of the parasite were then re-challenged with a lethal dose of *P. yoelii *17XL. Serum samples from GA cured mice that were re-challenged with *P. yoelii *17XL were examined for the presence of antibodies against the parasite proteins using western blot analysis.

**Results:**

Treatment of *P. yoelii *17XL infected mice with GA derivatives showed slow recovery from clinical symptoms of the disease. Blood smears from drug treated mice indicated a dominance of ring stage parasites when compared to controls. Although, *P. yoelii *preferentially invades normocytes (mature rbcs), in drug-treated animals there was an increased invasion of reticulocytes. Cured animals exhibited robust protection against subsequent infection and serum samples from these animals showed antibodies against a vast majority of parasite proteins.

**Conclusions:**

Treatment with GA derivatives blocked the transition from ring to trophozoite stage presumably by the inhibition of HSP90 associated functions. Persistence of parasite in ring stage leads to robust humoral immune response as well as a shift in invasion specificity from normocytes to reticulocyte. It is likely that the treatment with the water-soluble GA derivative creates an attenuated state (less virulent with altered invasion specificity) that persists in the host system, allowing it to mount a robust immune response.

## Background

A recent WHO factsheet lists that in 2008, there were about 225 million cases of malaria and nearly 800,000 deaths [1]. These deaths are largely due to *Plasmodium falciparum *infection among young children from sub-Saharan Africa. Estimates about the reported deaths due to malaria in other regions of the world are highly uncertain and are likely to be much greater than the documented ones [2]. Observation that the repeated exposures to parasite in endemic regions can lead to development of immunity has stimulated intensive efforts to search for protective antigens to develop vaccines [3,4]. In last half a century, a variety of strategies involving immunization with different stages of parasite has thus far not culminated in any successful vaccine [5]. At present, malaria is curable, but excessive and non-compliant use of anti-malarial drugs, have resulted in the emergence of drug resistance that has spread very rapidly, eliminating the effectiveness of some of these drugs to cure the disease (for example chloroquine) [6-10]. There is an urgent need to develop a new class of anti-malarials that can target pathways and processes distinct from the existing therapeutic agents. In the last decade, *Plasmodium *genome sequencing [11] has greatly increased the repertoire of potential drug targets and possibilities for structure based rational drug design approaches to explore and develop novel anti-malarials [12]. Meanwhile, time tested approaches of screening compound libraries in cellular assays have yielded very promising results [13].

A naturally occurring benzoquinone ansamycin compound, geldanamycin (GA) is a specific inhibitor of heat shock protein 90 (HSP90) [14,15] and is a potential anti-cancer agent [16,17]. As the life cycle of *Plasmodium *requires two different hosts of which one is poikilotherm and other is a homeotherm, it is not surprising that a significant fraction of parasite genome (~2%) is dedicated to molecular chaperones [18]. As heat shock proteins are critical for maintaining a functional complement of proteins in the parasite, proteins like HSP90, HSP70/HSP40 and other smaller HSPs have been the major drug targets for anti-malarials. The blockade of HSP90 function by geldanamycin (GA) has been reported to inhibit the growth of the malarial parasite *Plasmodium falciparum *in in vitro cultures [19-21]. Using synchronized cultures of *P. falciparum*, Bhanumathy et al. observed that the geldanamycin treatment (24 h) causes specific blockade of the transition from ring to trophozoite stage in the life cycle of the parasite [19]. On the contrary, Kumar et al. [20] reported that the treatment of an asynchronous culture of *P. falciparum *3D7 with geldanamycin resulted in inhibition of all intra-erythrocytic stages and the parasites were destroyed in a single developmental cycle. Such a death and disintegration led to the appearance of pyknotic bodies in the GA treated cultures [20]. Irrespective of these discrepancies, it is clear that GA is effective in inhibiting the growth of *P. falciparum *in in vitro cultures of chloroquine sensitive (strain 3D7) as well as resistant (strain W2) strains. Thus, it appears to be a good candidate to develop as a novel class of anti-malarial.

In past, attempts have been made to develop geldanamycin as an anti-cancer drug. However, due to its low aqueous solubility and high hepatotoxicity [22], efforts were directed towards development of more water soluble and metabolically stable derivatives of GA. A synthetic analogue of geldanamycin, 17-allylamino-17-demethoxygeldanamycin (17-AAG) has been through phase-I trials for cancer treatment [23]. This experimental drug was found to have acceptable levels of hepatotoxicity. The growing evidence regarding the potential for useful anti-malarial activity by these experimental therapeutic agents and their derivatives warrants continued pre-clinical evaluation. To date, there has been no experimental work reported on the evaluation of the efficacy of geldanamycin-derivatives in curing malaria in animal model systems. This investigation was undertaken to test the anti-malarial activity of 17-AAG and a highly water soluble geldanamycin derivative, 17-N-(3-(2-(-2(3-aminopropoxy)ethoxy)propyl)pent-4-ynamide-17-demethoxygeldanamycin (17-PEG-Alkyn-GA) in an animal model system.

## Methods

### Materials

Chloroquine phosphate was a kind gift from BDH Industries LTD., Mumbai, India. Protease inhibitor cocktail (cat no. P2714) was obtained from Sigma-Aldrich. Swiss mice (4-6 weeks old) were provided by the animal house facility at Tata Institute of Fundamental Research, Mumbai, India. All chemicals used were of Analar grade. HRP conjugated anti-mouse IgG was from Sigma-Aldrich.

### Synthesis of geldanamycin derivatives

All reagents and solvents were purchased from commercial sources and used without further purification. ^1^H NMR (300 and 500 MHz) and ^13 ^C NMR (75 MHz) spectra were recorded in CDCl_3 _solution on a 300 MHz spectrometer. Chemical shifts were referenced to δ 7.26 and 77.0 ppm for ^1^H and ^13 ^C spectra, respectively. High-resolution mass spectra were generated at the Purdue Mass Spectrometry Facility. Thin-layer chromatography (TLC) was performed on 250 *μ*M and 1000 *μ*M silica gel plates. Flash chromatography was run using RediSep normal-phase flash columns (230-400 mesh). Geldanamycin was isolated from fermentation of *Streptomyces hygroscopicus *var. *geldanus *that was provided by Dr. David Newman, NCI-Frederick. The production of geldanamycin was modified from a previously established method [14]. Briefly, 100 mL of production medium in 500 mL tribaffled flasks with silicon closures were inoculated with confluent oatmeal slants and agitated at 150 rpm in the dark at 28°C. Initial metabolite productions were monitored from days 4.0 to 7.0 for harvest using HPLC [24]. Harvest on day 5.5 generally achieved productions of 1.01 +/- 0.29 nmoles per 5 mL. A sample of 17-*N*-allyl-17-demethoxygeldanamycin (17-AAG) was prepared after the established procedure from geldanamycin [25].

### *N*-(3-(2-(2-(3-aminopropoxy)ethoxy)ethoxy)propyl)pent-4-ynamide (Alkyn-PEG-amine)

To a solution containing 1 g (10.2 mmol) of 4-pentynoic acid in 20 mL anhydrous CH_2_Cl_2 _was added 2.1 g (10.2 mmol) of N,N'-Dicyclohexylcarbodiimide (DCC) and 3.1 g (30.5 mmol) of triethylamine at 25°C under N_2_. The reaction mixture was stirred at 25°C for 10 min, and then 6.7 g (30.5 mmol) 3,3'-(2,2'-oxybis(ethane-2,1-diyl)bis(oxy))dipropan-1-amine in 20 mL anhydrous CH_2_Cl_2 _was added. The reaction mixture was stirred at 25°C for another 3 h. The solution was filtered and concentrated under reduced pressure. The residue was loaded into 80 g flash silica gel column eluting with two volumes of 95:5 CH_2_Cl_2_-methanol. The purified product was eluted using step gradients of 10:1:1 followed by 10:1:2 methanol:NH_4_OH:10% NH_4_OAC in H_2_O to yield alkyn-PEG-amine 2.6 g (85%) as a colorless sticky liquid. TLC (80:10:10 methanol: NH_4_OH:10%NH_4_OAc in H_2_O) followed by Ninhydrin staining showed R_f _= 0.37. ^1^H NMR (CDCl3): δ 1.32 (dt, 4H), 1.74 (m, 2H), 1.88 (m, 2H), 1.95 (m, 2H), 3.0 (t, 1H), 3.35 (t, 2H), 3.57 (s, 12H), 7.33 (s, 2H), 7.55, (s, 1H) Mass spectrum (300.2), *m/z *301.19 (M + H)^+^.

17- *N*-(3-(2-(2-(3-aminopropoxy)ethoxy)ethoxy)propyl)pent-4-ynamide -17-demethoxygeldanamycin (17-PEG-Alkyn-GA)

To a solution containing 390 mg (1.3 mmol) of Alkyn-PEG-amine in 8 mL anhydrous CH_2_Cl_2 _was added 81 mg (0.14 mmol) of GA at 25°C under N_2_. The reaction mixture was stirred at 25°C for 4 h and to the resulting solution 30 mL CH_2_Cl_2 _was added, and washed with three 10-mL portions of H_2_O, three 10-mL portion of saturated brine. The organic layer was dried (Na_2_SO_4_) and concentrated under diminished pressure. The residue was purified by chromatography on a flash column, Eluted with 98:2 methylene dichloride-methanol gave 17-PEG-Alkyn-GA as purple solid: yield 42 mg (36%); Mass spectrum, *m/z 851.03 *(M + Na)^+^(C_34_H_48_O_2 _requires 828.45). A complete set of ^1^H and ^13 ^C NMR data are provided as Additional file 1.

### Parasite culture, treatment of infected mice, stage specific distribution counts

A lethal mouse malarial parasite, *P. yoelli *17XL was cultured in six-week old Swiss mice and parasite infected red blood cells (PRBCs) were used for infecting fresh mice by intra-peritoneal injection (~10^6 ^PRBC). Parasitaemia was scored everyday by tail bleeding and preparing thin blood smears from infected mice. The infected blood smears were stained with Giemsa; about 300-400 RBCs were examined by microscopy and the infected erythrocytes were reported as the percent of the total. The pharmacological agents were dissolved in 10% DMSO or water and injected intra-peritoneal. The fractional distribution of various intra-erythrocytic asexual stages of parasites were determined by counting rings, trophozoites and schizonts and expressed in terms of percentage of total infected or parasitized RBCs (PRBC).

### Challenging malaria survivor mice after drug treatment and collection of serum from immune mice

The infected mice that survived the malaria after drug treatment (17-AAG, 17-Alkyn-PEG-GA and chloroquine) were allowed to recuperate for one month after parasite clearance. Each surviving mouse was re-challenged by injecting with ~10^6 ^*P. yoelii *17XL PRBCs and the parasites were allowed to grow. Thin blood smears were made every day to estimate percentage parasitaemia. Some of these mice did not show disease symptoms and cleared parasitaemia completely after 21 days of parasite infection. Approximately 0.1 to 1.0 ml of blood samples were collected using capillaries and allowed to clot for 30 min at room temperature and then subjected to centrifugation for 10 min at 3000 × g. The supernatant (serum) was collected and stored at -80°C until further analysis.

To obtain parasite sensitive serum, mice were injected with lower doses of parasite (~10^4^) to sustain the viability of mice. After 21 days of post-parasite injection, serum samples were prepared as mentioned above. Naïve serum was collected from fresh mice.

### Preparation of *Plasmodium yoelii *cells

*Plasmodium yoelii *cells were prepared as described earlier [26], with slight modification. Briefly, the mice were infected with *P. yoelii *17XL (lethal strain) and the parasites were allowed to grow until the infected red blood cells reached ≥ 30%. At this stage, 1-2 mL of blood was collected in equal volume of anti-coagulant solution (136 mM glucose, 42 mM citric acid and 75 mM Sodium citrate). Red blood cells (RBCs) were collected by centrifugation (1500 × g for 10 minutes) and washed three times with phosphate buffer saline (PBS) (137 mM NaCl, 2.7 mM KCl, 10 mM Na_2_HPO_4_, 1.8 mM KH_2_PO_4_, pH 7.4). The RBCs were re-suspended in PBS containing 1 mM PMSF and appropriate amounts of protease inhibitor cocktail as recommended by the supplier (Sigma-Aldrich). To this suspension of infected RBCs, 0.05% saponin was added and allowed to incubate for 1 min at 37°C. The solutions were then kept at room temperature (~20°C) for 30 minutes to release the parasite from the infected RBCs. Parasite cells were collected by centrifugation at 18000 × g for 10 min and the pellets washed with PBS to remove all the hemoglobin (as judged by red color). The cell pellet was stored at -80°C until further analysis.

### Preparation of parasite and RBC cell lysates

Parasite cell pellets (~200 μg) were suspended in 200 μL of PBS containing 5 mM EDTA, 1 mM PMSF and protease inhibitor cocktail (Sigma-Aldrich). After incubation on ice for 10 minutes, the cells were subjected to freeze-thaw (six cycles) by freezing in liquid nitrogen (2 minutes) and thawing at room temperature (2 minutes). These cells were then subjected to ultrasonification for 10 seconds at constant duty cycle by using Branson Sonifier 450 and then the sample was incubated on ice for 1 minute. This process was repeated six times. The cell extract was centrifuged at 100,000 × g for 30 minutes and the supernatant collected was the cytosolic fraction. Protein concentrations of the samples were estimated by measuring OD_280 nm_. To prepare RBC extract, ~1 mL blood was collected from mice with 0% (uninfected), ~3% or ~30% parasitaemia in equal volume of anticoagulant. RBCs were collected by centrifugation and were lysed by using 0.05% saponin as mentioned above. The supernatant obtained by centrifuging of the lysed RBCs at 14000 × g was collected as RBC extract. Protein concentration of sample extracts was measured at OD_280 nm_.

### SDS-PAGE and Western blotting

SDS-PAGE and Western blotting was performed as described earlier [27]. Typically 20 μg of cellular (parasite or RBC) protein extracts were analyzed using a 12% SDS-gel and visualized with silver stain or transferred to a PVDF membrane for Western blotting using Bio-Rad Trans-Blot Semi-Dry Transfer Cell. The blots were probed by using various anti-sera (1:1,000 dilution in 1× PBS), followed by secondary HRP conjugated anti-mouse IgG (Sigma-Aldrich) used at 1:1,000 dilutions.

## Results

### Effect of geldanamycin- derivatives on *P. yoelii *17XL growth in infected mice

**Figure 1:**
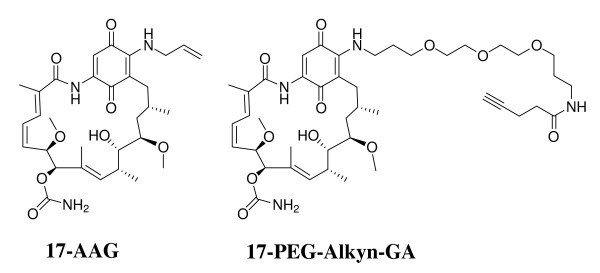
**Chemical structures of 17-N-Allylamino-17-demethoxygeldanamycin (17-AAG) and 17-pegylated-N-(4)-pentynoyl-GA (MW 828.45)**.

**Figure 2:**
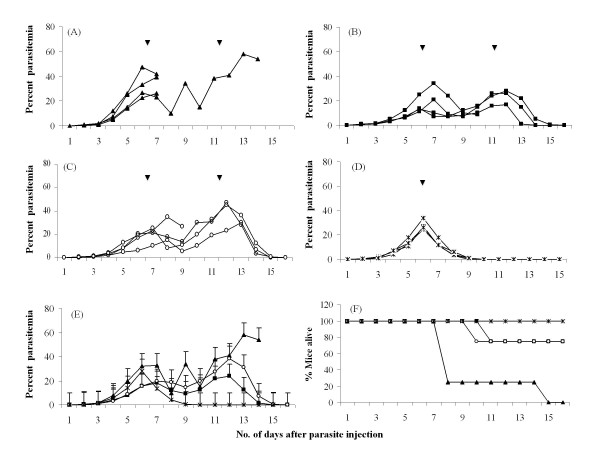
**Effect of 17-AAG and 17-PEG-Alkyn-GA treatment on parasite growth and mortality of *P. yoelii *17 XL infected mice**. Four groups (A-D) of mice were injected with ~10^6 ^*P. yoelii *17 XL infected mouse red blood cells and parasitemia was monitored every day. On days 6^th^ and 12^fth ^post-infection, mice were injected with: (A) 10% DMSO (control) (▲); (B) 300 nMoles of 17-AAG dissolved in 10% DMSO (■); (C) 300 nMoles of 17-PEG-Alkyn-GA dissolved in 10% DMSO (○); (D) 300 nMoles of chloroquine (in water and was injected on day 6^th ^only) (x); (E) average percent parasitemia for the four groups and (F) survival profile. There were 4 animals (n = 4) in each group.

Two different derivatives of geldanamycin, namely 17-allylamino-17-demethoxygeldanamycin (17 AAG) and a highly water soluble pegylated derivative of GA, 17-N-(3-(2-(-2(3-aminopropoxy)ethoxy)propyl)pent-4-ynamide-17-demethoxygeldanamycin (17-PEG-Alkyn-GA) (Figure 1) were tested on *P. yoelii *infected mouse malaria model system. Four groups (each group having four animals) of mice were infected with the parasite (intra-peritoneal injection of ~10^6 ^PRBCs). After 6 days when the average parasitaemia reached ~8-12% and all the animals displayed characteristic symptoms of malaria, the control group was injected with vehicle control (10% DMSO). For drug administration, 300 nmoles of each agent constituted a single dose. The second group of mice was injected with 300 nmoles of 17-AAG (MW 585.31; 0.18 mg/mouse/dose; 7.2 mg/Kg body weight) and the third group was injected with 300 nmoles of 17-PEG-Alkyn-GA (MW 828.45; 0.25 mg/mouse/dose; 10.2 mg/Kg body weight) (dissolved in 10% DMSO). The fourth group of mice was injected with 300 nmoles chloroquine phosphate dissolved in water. Parasitaemia was monitored every day until either the mice died or could clear the parasites. Figure 2(A-D) shows the parasitaemia profiles for control (untreated), 17-AAG, 17-PEG-Alkyn-GA and chloroquine treated mice. The arrowheads mark the 6^th ^and the 12^fth ^day when the drugs were injected. Figure 2E presents the average parasitaemia for different groups of mice. In control groups, the parasitaemia reached almost ~60% and all the animals died by day 14 post-infection (Figure 2F). Single dose treatment with chloroquine on 6^th ^day post infection was adequate to clear the parasites and cure the mice of the disease. Injection of GA derivatives on day 6 post-infection did result in control of parasitaemia. However, a second dose was needed for complete clearance of the parasites. Out of four animals in two groups treated with GA derivatives, one animal succumbed to infection. These data support the conclusion that geldanamycin derivatives can cure malaria. However, the current data indicates that the GA analogs are not very effective in a single dose (non-optimized) as compared to chloroquine.

### Treatment with geldanamycin derivatives caused the parasite to switch its invasion specificity from normocytes to reticulocytes

**Figure 3:**
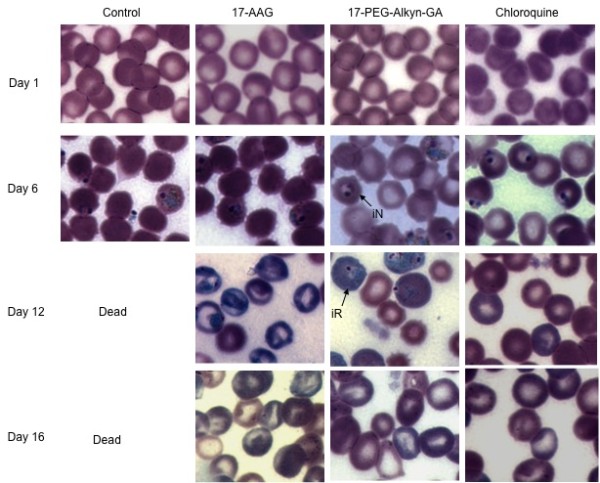
**Treatment of *P. yoelii *infected mice with geldanamycin (17-AAG and 17-PEG-Alkyn-GA) resulted in change in invasion specificity of the parasite from normocytes to reticulocytes**. Naïve mice were infected with *P. yoelii *and geimsa stained smears were examined everyday from each mice. After the mice became clinically sick, 17-AAG, 17-PEG-Alkyn-GA and chloroquine were injected (6^th ^& 12^fth ^days post infection) to three different groups (four animals in each group) of mice. Control group mice were not treated with any drug and only a single dose of chloroquine was injected. Representative blood smears are shown. Preferential invasion of reticulocytes was observed in 17-AAG and 17-PEG-Alkyn-GA treated groups of mice. iN, infected normocytes and iR, infected reticulocytes.

The blood smears that were prepared for each mouse on daily basis were examined for the evaluation of invasion of normocytes and reticulocytes. Before the injection of drugs (i.e. until the day 6 post-infection), parasitaemia was exclusively restricted to normocytes. Mice that were subjected to treatment with 17-AAG and 17-PEG-Alkyn-GA, preferential invasion of reticulocytes was observed. However, blood smears from control and chloroquine treated mice continued to show normocyte invasion until either the death of the animal (control group) or clearance of the parasites (chloroquine group) (Figure 3). Depending on the strain, *P. yoelii *is known to invade both, normocytes, as well as reticulocytes. Normocytic invasion is lethal while reticulocyte invasion is rather benign [28,29]. The *P. yoelii *17XL strain is known to invade normocytes [28] and the same is observed here. However treatment with GA-derivatives resulted in alteration of this specificity from normocytes to reticulocytes. It is likely that this change in invasion specificity renders the parasite benign and may contribute, in part to why the host system eventually succeeds in clearance of the pathogen.

### GA-derivative drugs block the progress of ring to trophozoite stage of the parasite

**Figure 4:**
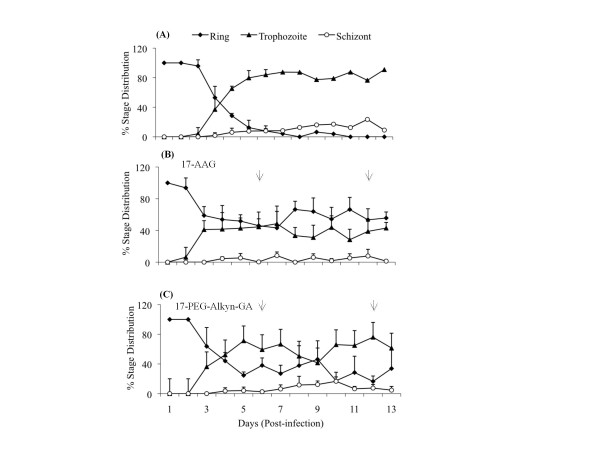
**Treatment with 17-AAG and 17-PEG-Alkyn-GA of infected mice showed blockage of ring to trophozoite transition**. Stage (ring, trophozoite or schizont) specific parasite count was made on all giemsa stained blood smears. Data about each stage are presented as percent of total infected cells. (A) Control, (B) 17-AAG treated and (C) 17-PEG-Alkyn-GA treated.

Daily blood smears prepared for each animal were also scored for fractional distribution of various intra-erythrocytic asexual stages (rings, trophozoites and schizonts) of the parasite. In each smear, rings, trophozoites and schizonts were counted and plotted as the percent of total infected cells. Data are presented in Figure 4. In the control group of mice, initially (1-3 days post infection), most parasites are in the ring stage. With time, there is a gradual decrease in the ring population with concomitant increase of trophozoites (Figure 4A), suggesting constant transitions of rings to trophozoites. However, on treatment with 17-AAG or 17-PEG-Alkyn-GA on day 6 post infection, population of rings increased and stabilized at much greater fraction (Figure 4B, day 7-10 post infection) as compared to the control group (Figure 4A). Such a distribution can arise if there is a blockade or reduction in transition from ring to trophozoite stage. The profile of stage specific distribution of the parasites was rather similar for the both the geldanamycin derivatives used (17-AAG or 17-PEG-Alkyn-GA). These observations are in agreement with the conclusions arrived at by Bhanumathy et al. [19] where it was demonstrated that GA blocks the progression of rings into trophozoites in in vitro cultures of *P. falciparum*. In the blood smears examined here, pycnotic bodies were never observed. Thus, it is unlikely that there is any large scale death and disintegration of the parasite in response to GA treatment as reported earlier [20].

### GA-derivative drug treated mice exhibit resistance to subsequent infection

**Figure 5:**
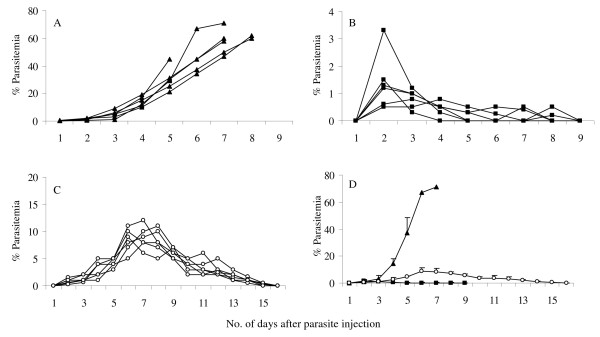
***P. yoelii *infected mice that were cured with geldanamycin derivatives exhibit robust immunity against subsequent infection**. Infection was induced by injecting ~10^6 ^*P. yoelii *infected mice rbcs. Response of infection was studied in four different groups of mice. (A) Naïve 10 weeks old Swiss mice (control) (▲); mice that were cured of malaria by treatment with (B) 17-AAG or 17-PEG-Alkyn-GA (■) and (C) chloroquine (○) (see Figure 1 for B and C groups of mice). (D) Average parasitemia profile of the three groups. Each group had 6 animals.

In GA-derivatives treated mice, parasite persisted for a prolonged period (Figure 2E) in the host and eventual clearance was through reticulocyte invasion. It is likely that such prolonged exposure to parasite may result in development of immunity to subsequent challenges of *P*. *yoelii*. To test this hypothesis, the mice that were cured by GA-derivative drug treatment were allowed to recover and live a healthy life for 30 days and then challenged with a fresh dose of *P. yoelii *17XL. For comparison, second group of mice that had been cured from malaria symptoms by treatment with chloroquine and allowed to recover for 30 days, were also challenged for the second time. To ensure that the parasite is lethal, a control set consisting of four fresh naïve mice was also included. Parasitaemia profiles in these three groups of mice were monitored daily and are shown in Figure 5. As expected control mice had high parasitaemia (40-60%) that resulted in their death between days 5 to 8 (Figure 5A). The group treated with geldanamycin derivatives showed very low parasitaemia (< 4%) that peaked on day 2 and got cleared by day 9 (Figure 5B). Chloroquine-treated mice had intermediate profile with parasitaemia reaching around 8-13% that did clear by day 16 (Figure 5C). Average parasitaemia profiles of these three groups are shown in Figure 5D. These results suggest that mice treated with HSP90 antagonists developed a robust immunity against subsequent challenge with the parasite. In order to examine the profile of antibodies generated in different groups of mice, serum samples were collected from each of these mice and pooled together for each group.

### Sera from 17-AAG and 17-PEG-Alkyn-GA treated mice have antibodies against multiple parasite proteins

**Figure 6:**
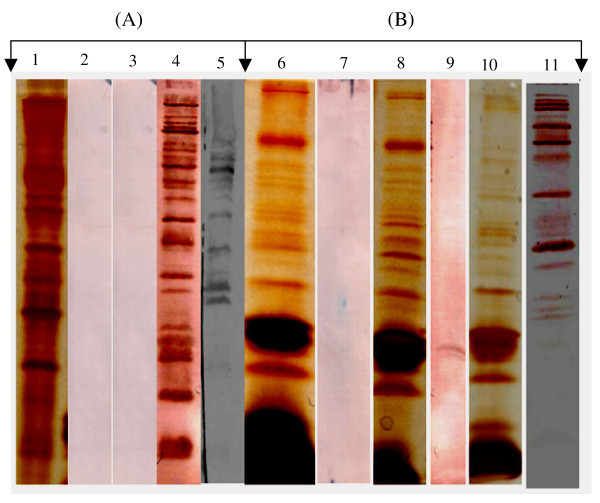
**Serum samples from geldanamycin treated mice have antibodies against vast majority of parasite proteins**. Protein extracts from (A) *P. yoelii *and (B) mouse rbcs were analyzed on 12% SDS-PAGE. Gels were either silver stained (lanes 1, 6, 8 & 10) or blotted on PVDF membrane and probed with various serum samples collected from mice. (A) *P*. *yoelii *cell extract: silver stained (lane 1); Western using naïve mice serum (lane 2); malaria sensitive mouse serum (lane 3); serum from GA treated mice (lane 4) and serum from chloroquine treated mice (lane 5). (B) Mouse RBC extract: un-infected (lanes 6 & 7), ~3% parasitemia (lanes 8 & 9) and ~30% parasitemia (lanes 10 & 11). Lanes 6, 8 & 10 are silver stained while lanes 7, 9 & 11 are western blots developed using pooled sera from GA treated mice.

To examine the antibody profiles of serum samples collected from above mentioned three groups of mice, proteins from the whole cell parasite extracts were separated using a 12% SDS-PAGE and transferred to a PVDF membrane. These blots were subjected to western analysis using different serum samples. Results of such western analysis are shown in Figure 6. Lane 1 is a silver stained protein profile of the whole cell *P. yoelii *extract. Western blots made using the sera collected from naïve (lane 2) and parasite sensitive mice sera (lane 3) did not show any reactivity towards the parasite proteins. In contrast to these, serum samples collected from 17-AAG or 17-PEG-Alkyn-GA treated mice had antibodies against vast majority of parasite proteins (lane 4). Sera collected from the chloroquine treated group showed antibodies against a subset of the parasite proteins (lane 5). These data are consistent with a hypothesis that drug-induced antibody response mounted by the host against the drug-attenuated parasite leads to protection against a subsequent parasite challenge.

## Discussion

Geldanamycin is a benzoquinone ansamycin antibiotic that exerts its pharmacological effects by binding to the ATP site of HSP90 and interfering with its chaperoning functions. HSP90 is a ubiquitous molecular chaperone critical for the folding, assembly and activity of the signaling proteins that promote the survival and the growth of dividing cells [17,30-33]. Binding of GA to HSP90 results in dissociation of chaperone-client protein complexes and induces the degradation of client proteins. It is believed that such destabilization of client proteins (like raf, Src, Lck, Wee1, Mek, Cdk4, Src, Ck2, Akt, ErbB2 etc.) is responsible for the anti-mitotic and anti-tumor activity of the drug. As geldanamycin is highly hepatotoxic, a less toxic derivative of geldanamycin, 17-AAG was tested in Phase-I clinical trials as an anti-tumor agent [34-36].

As homologs of mammalian HSP90 are present in most pathogens, there is a possibility of GA emerging as a broad spectral anti-parasitic agent. Effects of inhibiting the functional activity of HSP90 using geldanamycin have been investigated on few pathogens. In *Leishmania donovani*, it is known that transition from insect stage promastigote to pathogenic mammalian stage, the amastigote is triggered by the rise in ambient temperature. Inactivation of HSP90 by GA mimics the temperature-induced differentiation from promastigote to amastigote. However, GA treatment of cultured promastigotes induced a growth arrest [37,38]. Macro-filaricidal activity of GA against cat and dog filaria has also been reported [39]. Recent observations about the ability of GA to kill adult male and female worms of *Brugia malayi *(that causes lymphatic filariasis] and *Schistosoma japonicum *(that causes schistosomiasis) suggests possibilities of wider therapeutic potential of this drug [40]. GA resistant homolog of HSP90 has been reported in nematode *Caenorhabditis elegans *[41,42] raising the possibility for the quick emergence of resistance against the drug.

As mentioned earlier, anti-plasmodial activity of geldanamycin has been investigated using *Plasmodium *cultures [18-21]. In the experiments reported here, these studies have been extended to an animal model and tested the anti-malarial potential of this drug. The two derivatives of geldanamycin (17-AAG & 17-PEG-Alkyn-GA) that were tested here, show anti-malarial activity and injection of two doses of 300 nmoles each per mouse were sufficient to clear the parasites (Figure 2). Detailed examination of distribution of parasites in various intra-erythrocytic stages (rings, trophozoites and schizonts) in drug treated and untreated (control) mice showed that in the treated group ring stage parasite persists resulting in the fractional increase of rings as compared to trophozoite. Such a distribution can arise if the drug treatment blocks the transition from ring to trophozoite stage. Infected erythrocytes with the blocked ring stage parasites may eventually haemolyse, releasing the parasite in the host circulatory system. Immune response to such released parasites may result in robust antibody response that conferred immunity to subsequent parasite challenges. The ability of the geldanamycin to block stage transition in the parasite life cycle appears to be equivalent to immunization with an attenuated strain of a pathogen. Attenuated *Plasmodium *sporozoites prepared by irradiation [43] or genetic manipulation [44] are known to induce immunity. The sera collected from geldanamycin derivative-treated animals exhibited reactivity against most of the parasite proteins indicating a robust humoral response. Such sera have proved to be very useful reagent for the detection of unknown parasite proteins in analytical experiments.

For malaria vaccine development, efforts have been made to target liver, blood and/or sexual transmission stages using conventional vaccine approach of exposing the host to relevant antigens. A compilation of different antigen formulations and evaluations of field trials can be found at WHO site [45]. Despite these efforts, there is currently no licensed, effective malaria vaccine. It is clear that for the purpose of malaria elimination, vaccines with much better efficacies are required [46]. An emerging approach to counteract the immune-modulating effects of the parasite is to co-administer the antigens along with sub-optimal doses of immune-modulating anti-malarial drugs [47,48]. The approach involves administration of virulent *Plasmodium *with sub therapeutic dose of an anti-malarial sufficient to contain the growth of the parasite to prevent symptoms while allowing induction of a protective immune response [49,50]. Robust immunity observed here in GA treated mice against a lethal strain of *P. yoelii *suggests that this drug can be a potential candidate for co-administration with pathogen for the induction of immunity.

As mentioned above, clearance of parasite in geldanamycin treated mice showed sequential changes in infectivity from mature red blood cells to reticulocytes. This change in invasion specificity was also associated with loss of virulence and self-resolution of infection. Many host and parasite factors may influence such transitions between virulent and non-virulent states of the parasite. Genetic polymorphism involving a single amino acid substitution in *P*. *yoelii *erythrocyte binding-like protein (*Pyebl*) has been reported to be one such factor [51-53]. Similar changes in invasion specificity for *P. yoelii *(from mature rbcs to reticulocytes) were observed in experiments where immune protection conferred by *P. falciparum *enolase was investigated (unpublished data). As strong host mounted immune responses occurred in HSP90 inhibitor treated animals, it could be directly associated with the cause of preferential invasion of reticulocytes. It is possible that the observed change in host cell invasion specificity in response to geldanamycin treatment may have arisen due to a point mutation as reported earlier [52]. Since this change in invasion specificity (from normocytes to reticulocytes) of *P. yoelii *17XL occurred in all the drug treated mice, it is highly unlikely that it can be due to a mutation in *Pyebl *[52]. As this change in specificity of invasion is associated with the slow growth as well as loss of virulence in the parasite, it is expected that the expressed proteomes of the normocyte invading and the reticulocyte invading parasites may have significant differences. It may be interesting to compare expressed proteomes from these two states (normocyte invading and reticulocyte invading) of *P. yoelii *17XL to identify the molecular players that participate in determining the host cell invasion specificity and virulence.

## Abbreviations

DMSO: dimethyl sulfoxide; GA: geldanamycin; 17-AAG: 17-allylamino-17-demethoxygeldanamycin; 17-PEG-Alkyn-GA: 17-N-(3-(2-(-2(3-aminopropoxy)ethoxy)propyl)pent-4-ynamide-17-demethoxygeldanamycin; PRBC: parasite infected red blood cell.

## Competing interests

The authors declare that they have no competing interests.

## Authors' contributions

RM: Performed all the experiments and analyzed the data. ZDX: Synthesized 17-AAG and 17-PEG-Alkyn-GA. AKHW: Characterized the structure of 17-PEG-Alkyn-GA by NMR. VJD: Conceived the idea of synthesizing 17-PEG-Alkyn-GA, helped in drafting the manuscript. GKJ: Conceived the study, planned the experiments, interpreted the results and wrote manuscript. All authors read and approved the final manuscript.

## Supplementary Material

Additional file 1 **Structural characterization of 17-PEG-Alkyn-GA**. Click here for file
