# Supplementary material for: Anti-malarial activity of geldanamycin derivatives in mice infected with Plasmodium yoelii
Source: Malar J. 2012 Feb 23;11:54. doi: 10.1186/1475-2875-11-54 (PMC3361485; doi:10.1186/1475-2875-11-54)

**Additional File 1: Structural characterization of 17-PEG-Alkyn-GA.**

**Table 1:** ^1^H and ^13^C NMR Chemical Shifts (ppm) of 17-N-(3-(2-(-2(3-aminopropoxy)ethoxy)propyl)pent-4-ynamide-17-demethoxygeldanamycin (17-PEG-Alkyn-GA) and Correlations Observed through COSY, HSQC, and HMBC Experiments

| Carbon skeleton number | Proton type, number expected | Carbon/ heteroatom hybridization | Estimated shifts | | Proton shift observed, [splitting, J (Hz), integration] | Carbon shift as inferred from: **13C spectrum** (deduced), HMQC (direct correlation),*HMBC (indirect correlation)* |
| --- | --- | --- | --- | --- | --- | --- |
|  |  |  | Proton | Carbon |  |  |
| 1 | none | sp^2^ carbonyl | none | 169.1 | none | **171***-171.93* |
| 2 | none | sp^2^ alkene | none | 133.2 | none |  |
| 2’ | sp^3^ methyl. 3 | sp^3^ | 2.43 | 12.5 | 2.02 (s, 3H) |  |
| 3 | sp^2^ alkene, 1 | sp^2^ alkene | 7.13 | 128.4 | 6.94 (d, J = 5.9, 1H) | **124** |
| 4 | sp^2^ alkene, 1 | sp^2^ alkene | 6.47 | 125.7 | 6.52 (dd, J = 11.8, 6.6, 1H), | 120.55-*122.62* |
| 5 | sp^2^ alkene, 1 | sp^2^ alkene | 5.84 | 137.8 | 5.85 (t, J = 10.5, 1H) | **102** |
| 6 | sp^3^ methine. 1 | sp^3^ | 4.15 | 85.3 | 6.47 (dd, J = 7.8, 5.1, 1H) | *98.038* |
| 6’ | sp^3^ methoxy, 3 | sp^3^ methoxy | 3.30 | 57.3 | 3.36 (s, 3H) | 52.228-*54.783* |
| 7 | sp^3^ methine. 1 | sp^3^ | 5.00 | 80.6 | 6.40 (dq, J = 14.2, 4.9, 1H), |  |
| 7’ | none | sp^2^ carbonyl | none | 156 | none | **156.3** |
| 7" | amide, 2 | sp^2^ amide | 7.68 | none | 4.31 (d, J = 10.0, 1H) |  |
| 8 | none | sp^2^ alkene | none | 132.6 | none | **133.8** |
| 8’ | sp^3^ methyl. 3 | sp^3^ | 1.82 | 13.4 | 1.79 (s, 3H) |  |
| 9 | sp^2^ alkene, 1 | sp^2^ alkene | 5.41 | 131.9 | 5.91 (d, J = 9.4, 1H) | **132.5** |
| 10 | sp^3^ methine. 1 | sp^3^ | 2.42 | 32.1 | 2.79 (dd, J = 14.4, 10.5, 1H) |  |
| 10’ | sp^3^ methyl. 3 | sp^3^ | 1.11 | 23.3 | 0.99 (d, J = 6.9, 3H), |  |
| 11 | sp^3^ methine. 1 | sp^3^ | 3.56 | 71.9 | 2.21 (dd, J = 2.9, 0.8, 1H) |  |
| 11’ | sp^3^ hydroxyl, 2 | sp^3^ hydroxyl | 3.58 | none | 4.62 (s, 1H) |  |
| 12 | sp^3^ methine. 1 | sp^3^ | 2.91 | 83.9 | 2.66 (d, J = 13.6, 1H) |  |
| 12’ | sp^3^ methoxy, 3 | sp^3^ methoxy | 3.30 | 57.4 | 3.26 (s, 3H) |  |
| 13 | sp^3^ methylene. 2 | sp^3^ | 1.50, 1.25 | 31 | 2.76-2.70 (m, 2H) |  |
| 14 | sp^3^ methine. 1 | sp^3^ | 1.69 | 26.3 | 1.69 (d, J = 3.5, 1H) |  |
| 14’ | sp^3^ methyl. 3 | sp^3^ | 0.96 | 18.4 | 0.96 (d, J = 6.6, 3H) |  |
| 15 | sp^3^ methylene. 2 | sp^3^ | 2.04, 1.79 | 32.6 | 2.48 (dd, J = 13.4, 11.8, 2H) |  |
| 16 | none | sp^2^ | none | 113.3 | none | **113** |
| 17 | none | sp^2^ | none | 146.5 | none | **144** |
| 18 | none | sp^2^ carbonyl | none | 181.7 | none | **184** |
| 19 | sp^2^ alkene, 1 | sp^2^ | 6.21 | 106.0 | 6.95 (s, 1H), | 106 |
| 20 | none | sp^2^ | none | 139.6 | none | **141** |
| 21 | none | sp^2^ carbonyl | none | 178.5 | none | **180** |
| 22 | amide 1 | sp^2^ nitrogen | *8.0-rough* | none | 9.19 (s, 1H) |  |
| 23 | amine, 1 | sp^3^ nitrogen | *2.0-rough* | none | 5.18 (s, 1H) |  |
| 24 | sp^3^ methylene. 2 | sp^3^ | 2.87 | 40.3 | 3.46 (d, J = 8.8, 2H) |  |
| 25 | sp^3^ methylene. 2 | sp^3^ | 1.73 | 30.4 | 1.78 (d, J = 5.9, 4H) (with C35) |  |
| 26 | sp^3^ methylene. 2 | sp^3^ | 3.37 | 66.3 | 3.57 (d, J = 5.3, 4H) (with C34) |  |
| 27 | none | sp^3^ oxygen | none | none | none |  |
| 28 | sp^3^ methylene. 2 | sp^3^ | 3.54 | 70.4 | 2.38 (td, J = 7.3, 4.9, 4H) (with C32) | 10.6 |
| 29 | sp^3^ methylene. 2 | sp^3^ | 3.54 | 70.4 | 2.51 (td, J = 7.3, 2.5, 4H) (with C31) | 30.7 |
| 30 | none | sp^3^ oxygen | none | none | none | 30.7 |
| 31 | sp^3^ methylene. 2 | sp^3^ | 3.54 | 70.4 | 2.51 (td, J = 7.3, 2.5, 4H), (with C29) | 10.6 |
| 32 | sp^3^ methylene. 2 | sp^3^ | 3.54 | 70.4 | 2.38 (td, J = 7.3, 4.9, 4H) (with C28) |  |
| 33 | none | sp^3^ oxygen | none | none | none |  |
| 34 | sp^3^ methylene. 2 | sp^3^ | 3.37 | 65.9 | 3.57 (d, J = 5.3, 4H) (with C26) |  |
| 35 | sp^3^ methylene. 2 | sp^3^ | 1.73 | 29.8 | 1.78 (d, J = 5.9, 4H) (with C25) |  |
| 36 | sp^3^ methylene. 2 | sp^3^ | 3.13 | 38.0 | 3.39-3.37 (m, 2H) |  |
| 37 | amide, 1 | sp^2^ nitrogen | 8.03 | none |  |  |
| 38 | none | sp^2^ carbonyl | none | 173.3 | none | **168.42** |
| 39 | sp^3^ methylene. 2 | sp^3^ | 2.35 | 32.7 | 3.72 (t, J = 4.8, 2H) |  |
| 40 | sp^3^ methylene. 2 | sp^3^ | 2.31 | 18.4 | 3.68 (t, J = 4.5, 2H) |  |
| 41 | none | sp alkyne | none | 83.8 | none | **73** |
| 42 | terminal alkyne | sp alkyne | 2.83 | 69.7 | 2.08 (s, 1H) | **80** |

**Table 2:** NMR Parameters for 17-N-(3-(2-(-2(3-aminopropoxy)ethoxy)propyl)pent-4-ynamide-17-demethoxygeldanamycin (17-PEG-Alkyn-GA)

1-H NMR (500 MHz; CDCl3): δ 9.19 (s, 1H), 6.95 (s, 1H), 6.94 (d, J = 5.9, 1H), 6.52 (dd, J = 11.8, 6.6, 1H), 6.47 (dd, J = 7.8, 5.1, 1H), 6.40 (dq, J = 14.2, 4.9, 1H), 5.91 (d, J = 9.4, 1H), 5.85 (t, J = 10.5, 1H), 5.18 (s, 1H), 4.62 (s, 1H), 4.31 (d, J = 10.0, 1H), 3.72 (t, J = 4.8, 2H), 3.68 (t, J = 4.5, 2H), 3.57 (d, J = 5.3, 4H), 3.46 (d, J = 8.8, 2H), 3.39-3.37 (m, 2H), 3.36 (s, 3H), 3.26 (s, 3H), 2.79 (dd, J = 14.4, 10.5, 1H), 2.76-2.70 (m, 2H), 2.66 (d, J = 13.6, 1H), 2.51 (td, J = 7.3, 2.5, 4H), 2.45 (dd, J = 13.4, 11.8, 2H), 2.38 (td, J = 7.3, 4.9, 4H), 2.21 (dd, J = 2.9, 0.8, 1H), 2.08 (s, 1H), 2.02 (s, 3H), 1.79 (s, 3H), 1.78 (d, J = 5.9, 4H), 1.69 (d, J = 3.5, 1H), 0.99 (d, J = 6.9, 3H), 0.96 (d, J = 6.6, 3H)

13-C NMR (500 MHz; CDCl3): δ 183.912, 180.268, 170.791, 168.489, 156.258, 145.177, 141.358, 135.639, 134.979, 132.648, 129.273, 126.780, 126.686, 108.718, 108.165, 99.4844, 83.0885, 81.3835, 72.6179, 70.6424, 70.5108, 70.4352, 70.3641, 70.2598, 70.0384, 69.5499, 69.1419, 68.6926, 57.1222, 56.7110, 53.4475, 44.7677, 38.4215, 34.4265, 32.2371, 29.1528, 28.6684, 28.2099, 22.8966, 21.2749, 20.9442, 20.6111, 20.5482, 18.8531, 18.3836, 17.4274, 14.8934, 12.7934, 12.6601

**Table 3** Peak intensities of ^13^C spectrum

line ppm Hz intensity

001 183.9128 23119.91 -1222081

002 180.2681 22661.72 -1225973

003 170.7910 21470.34 -1269840

004 168.4896 21181.04 -1271529

005 156.2585 19643.44 -1261439

006 145.1773 18250.42 -1190260

007 141.3584 17770.34 -1161775

008 135.6393 17051.38 -1093947

009 134.9794 16968.43 -1089394

010 132.6483 16675.38 -1060402

011 129.2737 16251.16 -1034834

012 126.7804 15937.72 -967841

013 126.6860 15925.85 -1009371

014 108.7187 13667.17 -769610

015 108.1651 13597.57 -757302

016 99.4844 12506.31 -653425

017 83.0885 10445.16 -453740

018 81.3835 10230.82 -390024

019 72.6179 9128.89 -301305

020 70.6424 8880.55 45123

021 70.5108 8864.00 5472

022 70.4352 8854.49 7072

023 70.3641 8845.56 112870

024 70.2598 8832.44 228331

025 70.0384 8804.61 173257

026 69.5499 8743.20 -183672

027 69.1419 8691.91 85010

028 68.6926 8635.43 -273205

029 57.1222 7180.90 203589

030 56.7110 7129.22 96165

031 53.4475 6718.95 893200

032 44.7677 5627.81 -66096

033 38.4215 4830.01 55711

034 34.4265 4327.80 124807

035 32.2371 4052.57 165596

036 29.1528 3664.83 310436

037 28.6684 3603.95 524719

038 28.2099 3546.30 194033

039 22.8966 2878.36 245181

040 21.2749 2674.49 212242

041 20.9442 2632.92 196448

042 20.6111 2591.05 224077

043 20.5482 2583.13 219964

044 18.8531 2370.05 228879

045 18.3836 2311.02 244675

046 17.4274 2190.82 366012

047 14.8934 1872.27 763737

048 12.7934 1608.28 342239

049 12.6601 1591.52 463359

**Determination of spin-systems**

*C1-C6*

The resonance at 6.94 ppm was assigned to the C-3 proton and the resonance at 6.52 ppm was assigned to the adjacent proton on C-4. A strong COSY correlation was observed and a coupling constant between 5.8 and 6.6 Hz was observed. The resonance at 6.52 ppm was also coupled to the resonance at 5.85 ppm in the COSY and had a second coupling constant of 11.8 Hz indicating a cis-relationship on the olefin. The resonance at 5.85 ppm additionally coupled to a resonance at 6.47 ppm, which in turn coupled to the proton at 6.40 ppm. These resonances were assigned as the methine protons on the next carbons bearing the methoxy and carbamate, respectively.

*C9-C15*

Two aliphatic methyl groups were observed at 0.99 and 0.96 ppm. The COSY showed strong cross peaks between 0.99 ppm and a 1-proton resonance at 2.79 ppm as well as a cross-peak to 1.69 ppm for the 0.96 resonance. The 0.99 and 2.79 pair was assigned to the carbon between the 8-9 alkene and the hydroxyl-bearing C-11 carbon because of coupling to the resonance at 5.91 ppm. This left the 0.96 and 1.69 ppm resonances for the carbon at position at C-14. The proton at 1.69 ppm coupled to resonances at 2.73 and 2.48 ppm. This was reasonable as the flanking protons were both methylenes. The resonance at 2.48 was showed coupling to a 1-proton resonance at 2.66. Initially the resonance at 2.48 was assigned to the C13 position as the 2.66 ppm resonance would be the C-12 methine. The resonance at 2.48 ppm was then reassigned to the C15 position because its 1D proton shifts were clearer than the resonance at 2.73, indicating a less-crowded environment as found at C-15. The coupling to the resonance at 2.66 could be rationalized as a through-space or perhaps a 5-bond coupling between the respective protons.

*C24-C36 – PEG linker*

The COSY spectrum showed a strong cross-peak between proton resonances at 2.51 and 2.38. Each of these peaks was a triplet-of-doublets and integrated for 4 protons each. These resonances were assigned to the inner ethylenes of the PEG-linker. An additional section of cross peaks were found at shifts of 3.57, 3.46, 3.38, and 1.78. The resonance at 1.78 integrated for 4 protons and was assigned to the two central carbons of the two propylene groups of the PEG-linker as these would be the least downfield resonances. The resonance at 3.57 ppm additionally integrated for 4 protons and was assigned as identical methylene groups on the three-carbon spacers on the PEG chain. The innermost methylene group of the three-carbon spacer was chosen as the location of these protons as the innermost pairs of protons would have more similar chemical and magnetic environments than the outermost pair. Additionally, this idea was supported by the observed pairwise shifts of all the nearby methylene groups. The two remaining resonances at 3.46 and 3.38 each integrated for two protons and were assigned to the outermost pair of methylenes. The resonance at 3.46 was assigned to the amine nearest the quinone ring as it should be more deshielded by the ring system compared to the amide at the end of the linker which was assigned the upfield resonance.


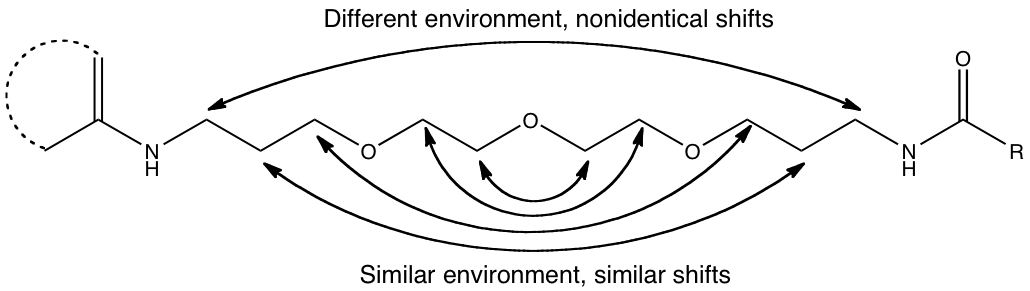

Supplement: Additional file 1 — Structural characterization of 17-PEG-Alkyn-GA. [file 1475-2875-11-54-S1.DOCX]
